# Mycorrhiza Symbiosis Increases the Surface for Sunlight Capture in *Medicago truncatula* for Better Photosynthetic Production

**DOI:** 10.1371/journal.pone.0115314

**Published:** 2015-01-23

**Authors:** Lisa Adolfsson, Katalin Solymosi, Mats X. Andersson, Áron Keresztes, Johan Uddling, Benoît Schoefs, Cornelia Spetea

**Affiliations:** 1 Department of Biological and Environmental Sciences, University of Gothenburg, Box 461, 405 30 Gothenburg, Sweden; 2 Department of Plant Anatomy, Eötvös Loránd University, H-1117 Budapest, Hungary; 3 Mer, Molécules, Santé, MicroMar—EA2160, LUNAM Université, IUML – FR 3473 CNRS, University of Le Mans, 72085 Le Mans Cedex 9, France; University of California—Davis, UNITED STATES

## Abstract

Arbuscular mycorrhizal (AM) fungi play a prominent role in plant nutrition by supplying mineral nutrients, particularly inorganic phosphate (P_i_), and also constitute an important carbon sink. AM stimulates plant growth and development, but the underlying mechanisms are not well understood. In this study, *Medicago truncatula* plants were grown with *Rhizophagus irregularis* BEG141 inoculum (AM), mock inoculum (control) or with P_i_ fertilization. We hypothesized that AM stimulates plant growth through either modifications of leaf anatomy or photosynthetic activity per leaf area. We investigated whether these effects are shared with P_i_ fertilization, and also assessed the relationship between levels of AM colonization and these effects. We found that increased P_i_ supply by either mycorrhization or fertilization led to improved shoot growth associated with increased nitrogen uptake and carbon assimilation. Both mycorrhized and P_i_-fertilized plants had more and longer branches with larger and thicker leaves than the control plants, resulting in an increased photosynthetically active area. AM-specific effects were earlier appearance of the first growth axes and increased number of chloroplasts per cell section, since they were not induced by P_i_ fertilization. Photosynthetic activity per leaf area remained the same regardless of type of treatment. In conclusion, the increase in growth of mycorrhized and P_i_-fertilized *Medicago truncatula* plants is linked to an increase in the surface for sunlight capture, hence increasing their photosynthetic production, rather than to an increase in the photosynthetic activity per leaf area.

## Introduction

Arbuscular mycorrhizal (AM) symbiosis is estimated to occur in 80% of the plant species and more than 90% of the cultivated species [1,2]. The current hypothesis is that development of the AM symbiosis played a crucial role in the initial colonization of land by plants and in the evolution of vascular plants [3,4]. AM fungi are obligate symbionts from the phylum Glomeromycota [5]. In controlled growth experiments, almost any AM fungal species or strain can be combined with most plant species allowing for establishment of the AM symbiosis [6] due to the presence of mycorrhizal genes in the common ancestor of plants [1]. The model legume *Medicago truncatula (M. truncatula*) and several crop plants (*e.g*., maize, sorghum, and soybean) are often used as host partners when studying the AM symbiosis [7].

Mycorrhization stimulates plant growth based on three main functions of the AM fungi: (*i*) stimulation of plant development by impacting the phytohormone balance; (*ii*) enhancement of plant fitness by increasing resistance or tolerance to biotic and abiotic stress; and (*iii*) improvement of plant nutrition by supplying mineral nutrients, particularly inorganic phosphate (P_i_) [2]. In exchange, the plant transfers the carbohydrates glucose and fructose to the intraradical hyphae, where they are converted to trehalose and lipids, the latter being translocated to the extraradical mycelium for further metabolism [8]. It is assumed that the total carbohydrate cost of the AM symbiosis can be up to 20% of the host plant photosynthetic production [9], but this varies greatly depending on the AM fungal species [10,11]. The increased sink strength is balanced by greater photosynthetic CO_2_ fixation in the source leaves of mycorrhized plants (*e.g*., [12]). However, it is not yet resolved whether enhanced carbon assimilation is caused by improved nutrient supply, by the increased carbon drain of mycorrhized roots or by an alternative mechanism.

The photosynthetic process providing carbohydrates for the fungal partner takes place in leaf chloroplasts. To our knowledge, there are no studies on the effect of mycorrhization on the chloroplast ultrastructure and its membrane-lipid composition. While the effects of AM fungal colonization on CO_2_ fixation in leaves have been analysed many times before, the impact on leaf chlorophyll (Chl) content and fluorescence is less well examined, and the described results appear to be contradictory. Many reports describe stimulatory effects [13–16], whereas Boldt et al. [12] reported a decrease, and Parádi et al. [17] found no effect of mycorrhization on Chl content and fluorescence. The reason for this discrepancy remains unclear as well as the mechanism by which AM could influence these photosynthetic parameters in plant leaves.

The positive effects of AM on lateral root formation and branching are well-documented, and the mechanisms involving fungal exudates, changes in P_i_ content, phytohormones and delivery of sugars have been recently reviewed [18]. The effects on shoot branching are less studied in mycorrhized plants, and thought to involve light in addition to nutrient and phytohormone levels [18]. *M. truncatula* shoots have a complex branched architecture due to the presence of two types of growth axes (main and axillary axes). Several models and several nomenclatures have been established to describe the vegetative and reproductive developmental stages in detail [19,20]. Bucciarelli et al. [19] identified several morphological differences in *M. truncatula* shoots resulting from growth under P_i_-deprivation conditions, which could be relevant in the context of the present work. In that study, plants grown under optimal P_i_ conditions showed earlier leaf development and expansion along the main and axillary axes of growth, earlier axillary shoot emergence and elongation, and an increase in leaf and shoot size as compared to P_i_-deprived plants. To our knowledge, the effects of mycorrhization on shoot development and leaf anatomy have not been studied in *M. truncatula*. In addition, it has not been investigated whether the effects are similar or distinct from those active in P_i_-fertilized plants. Most studies on the shoot of mycorrhized *M. truncatula* were performed at a certain plant age and level of root colonization (*e.g*., [21,22]). In this work, we hypothesized that AM stimulates the growth of *M. truncatula* through either modifications of leaf anatomy and/or photosynthetic activity per leaf area. We investigated whether these effects are shared with P_i_ fertilization in the absence of mycorrhization, and also assessed the relationship between levels of AM colonization and these effects.

## Materials and Methods

### Plant material, treatments and growth conditions

*M. truncatula* cv. Jemalong line J5 was used in this study. The cultivation procedures, growth conditions and nutrient solutions were based on previously published protocols [23–25]. Seeds were scarified with sandpaper, surface sterilized, and then germinated on 0.7% Bacto-agar plates in darkness at room temperature for four days. Seedlings were transplanted into 450 ml plastic pots containing Agsorb (24/48 LVMGA, Oil-Dri Corporation, Chicago, IL, USA) as substrate and 30–40% inoculum and grown for eight weeks *post* inoculation (wpi). The AM fungus *Rhizophagus irregularis (syn. Glomus intraradices)* BEG141 [26] was grown in pot cultures with leek (*Allium porrum*) for ten weeks. AM inoculum consisted of Agsorb and colonized roots from the leek. An inoculum composed of Agsorb and roots from leek that has been grown in the absence of fungus was used for cultivation of control and P_i_-fertilized (P_i_) plants. This type of inoculum allowed us to exclude the contribution from the presence of leek roots to the effects observed in the AM plants. It is of note that this type of inoculum is distinct from those found in the literature using mixture of sand and fresh soil (*e.g.*, [22]), or where a bacterial filtrate of the inoculum was used to exclude effects from AM-associated bacteria (*e.g.*, [12,27]). The *M. truncatula* plants were grown using a 16-h light (350–400 μmol photons m^−2^ s^−1^, 25–26°C, 30–47% relative humidity) and 8-h dark cycle (20°C, 53–57% relative humidity) in a growth chamber (CLF PlantMaster, Plant Climatics GmbH, Wertingen, Germany). To inhibit nodulation with nitrogen-fixing bacteria, the plants were watered twice per week with a Long Ashton Nutrient Solution (Medicago-LANS), containing a double concentration of nitrate and with no added P_i_ [28,29]. For P_i_ fertilization, 5 mM NaH_2_PO_4_ was added to the modified Medicago-LANS solution.

### Mycorrhization

An ink and vinegar protocol [30] was adapted to stain the fungal structures in the roots. For each plant, 30 randomly chosen 1-cm long root fragments were examined at 100–200× magnification using differential interference contrast microscopy (DIC or Nomarski) on a Zeiss Axioplan 2 (Carl Zeiss Group, Oberkochen, Germany) or light microscopy on a Nikon Alphaphot-2 YS2 (Nikon, Tokyo, Japan). The degree of mycorrhizal colonization was estimated according to Trouvelot et al. [31]. From these estimates, the intensity of colonization (*M*%), the frequency of colonization (*F*%) and the arbuscules abundance (*A*%) were calculated. Two experiments were performed, for which the mycorrhization parameters are given in Tables 1 and 2, respectively.

**Table 1:** Intensity (*M*%), frequency (*F*%) of fungal colonization and arbuscules abundance (*A*%) in the roots of mycorrhized *Medicago truncatula*.

**wpi**	**2**	**3**	**4**	**5**	**6**	**8**
*M*%	86 ± 4^a^	86 ± 5^a^	80 ± 5^ab^	86 ± 5^a^	80 ± 11^ab^	72 ± 11^b^
*F*%	100 ± 0^a^	99 ± 13^a^	96 ± 13^a^	98 ± 14^a^	99 ± 1^a^	99 ± 1^a^
*A*%	56 ± 3^ab^	59 ± 9^a^	50 ± 9^ab^	49 ± 6^ab^	42 ± 15^bc^	28 ± 4^c^

The data are means of four plants ± SD. Values with different letters in each row are significantly different from each other according to one-way ANOVA followed by Student-Newman-Keuls test (*P*<0.05). wpi, weeks *post* inoculation.

**Table 2:** Anatomical features of leaves from control, mycorrhized (AM) and phosphate-fertilized plants (P_i_).

**Parameter**	**wpi**	**Control**	**AM**	**P_i_**
Leaflet area (cm^2^)	3	1.3± 0.2^a^	2.0 ± 0.2^b^	2.2 ± 0.3^b^
	5	1.6 ± 0.2^a^	1.9 ± 0.1^b^	2.7 ± 0.5^c^
Leaflet thickness (μm)	3	228 ± 14^a^	257 ± 43^b^	266 ± 13^b^
	5	231 ± 46^a^	251 ± 43^b^	239 ± 54^ab^
Palisade cell number/100 μm leaflet section	3	9.9 ± 1.7^a^	10.0 ± 1.2^a^	9.5 ± 1.0^a^
	5	10.1 ± 1.4^a^	10.3 ± 1.1^a^	9.9 ± 1.5^a^
Spongy cell number/100 μm leaflet section	3	9.8 ± 1.9^a^	9.2 ± 1.5^a^	9.4 ± 1.5^a^
	5	9.8 ± 2.0^a^	9.9 ± 1.3^a^	9.4 ± 2.4^a^
Number of chloroplasts/palisade cell section	3	8.4 ± 1.9^a^	9.6 ± 2.0^b^	9.2 ± 2.0^ab^
	5	8.6 ± 2.5^a^	10.0 ± 2.4^b^	8.7 ± 1.9^a^
Number of chloroplasts/spongy cell section	3	7.4 ± 1.5^a^	8.4 ± 2.0^b^	7.8 ± 2.3^ab^
	5	7.6 ± 2.0^a^	7.8 ± 2.1^a^	7.5 ± 1.9^a^

The data are means of four plants ± SD. Values with different letters in each row are significantly different across treatments according to Kruskal-Wallis non-parameteric ANOVA followed by Mann-Whitney rank-sum test (*P*<0.05). Mycorrhization conditions were as follows: *M*% 30±7, *F*% 62±7, *A*% 17±5 (at 3 wpi) and *M*% 90±2, *F*% 100±0, *A*% 53±8 (at 5 wpi). wpi, weeks *post* inoculation.

### Phosphorus, nitrogen and carbon content

Dry shoot material was ground in a Mixer Mill (MM 301, Retsch, Haan, Germany). Following oxidation of plant phosphorus (P) to P_i_ [32], the P_i_ content was determined by colorimetric analysis using ammonium-molybdate [33]. Nitrogen (N) and carbon (C) content of ground dry powder from shoots was measured using a CHNS analyzer (EA1108, Fison Instrument, Ipswich, UK).

### Metamer analysis

To characterize development, a simplified numbering system based on previous work on *M. truncatula* [19,20] was created. This species has a main axis (MA) with axillary axes (branches) of different orders (Bx). The MA and branches are made up of so-called metamers, each consisting of an internode stalk, a leaf and an axillary meristem (producing branches or reproductive organs). The appeared metamers on MA and on each primary branch (B1 to B7) were counted. In our study, a metamer was defined as appeared when the leaf bud was clearly visible and as fully developed when the leaf was fully unfolded/open and had a blue-green colour. For comparison, Moreau *et al.* [20] defined leaf appearance at the stage when the leaf was fully unfolded. The total number of metamers for each growth axis was plotted as a function of age expressed as days *post* inoculation (dpi). The initial rate of metamer appearance on each branch (*R_M_*) was calculated from linear regression of all time points of each curve. To estimate the appearance date of each growth axis, the x-intercept was calculated after linearization of the data using the equation ln(y) = ln(*a*) + *b* ln(x), except in the case of MA for which the equation y = a + *b* ln(x) was used, where *a* is a constant and *b* is a factor indicating the rate at which metamers were formed (see Results for details).

### Microscopic analyses

To study leaf anatomy, 2×1 mm sections from the central leaflet of four different leaves for each treatment were prepared for fixation as described [34]. Semi-thin sections were cut, stained with toluidine blue and observed with an Olympus BH2-RFCA light microscope (Olympus, Tokyo, Japan) equipped with Nikon COOLPIX 950 (Nikon, Tokyo, Japan) digital camera. Using the same magnification and a stage micrometer, leaflet thickness was determined for 2–3 different sections for each studied leaflet, and these data were used to calculate the average leaf thickness per treatment. The thickness values of the palisade and spongy parenchyma layers were determined in a similar manner for each treatment; areas containing vascular bundles were not used for the calculations. To compare cell numbers, palisade and spongy parenchyma cells were counted within 100 μm distances (windows) on leaflet sections. For each leaflet, 20 windows were studied and averaged, and these means were averaged per treatment. The number of chloroplasts was determined in individual palisade or spongy parenchyma cell sections. Values obtained for approx. 50 randomly chosen palisade and 50 spongy cell sections were first averaged for each leaf, and then these means were averaged per treatment. Therefore, the obtained values represent the average number of chloroplasts visible in cell sections, and do not correspond to the total chloroplast number per cell.

To study chloroplast ultrastructure, ultrathin sections were prepared as described [34], and investigated with a Hitachi 7100 transmission electron microscope (Hitachi, Tokyo, Japan) at 75 kV accelerating voltage. Chloroplast size (*i.e.*, maximal length and width of median chloroplast sections) was measured on approx. 100–150 randomly chosen chloroplasts from approx. 35–60 palisade and 50–60 spongy parenchyma cells in each of the studied leaves. The length/width ratio was determined for each chloroplast. The chloroplast data were averaged for each leaf, and then these mean data obtained for each of the four different leaves were averaged again to calculate the mean chloroplast dimension per treatment.

### Photosynthetic analyses

Leaf Chl and carotenoids were extracted in methanol as described [35]. Chl fluorescence was measured using a Pocket PEA (Hansatech Instruments Ltd, Norfolk, England), and maximum quantum yield of photosystem II (*F_v_*/*F_m_*) was calculated according to [36]. CO_2_ assimilation was measured on the central leaflet with a leaf gas-exchange instrument (LI-6400XT, LiCOR, Lincoln, Nebraska, USA). Responses of net CO_2_ assimilation rate (*A_n_*) to the leaf intercellular CO_2_ concentration (*C_i_*), so called *A_n_/C_i_* curves were recorded at saturating light intensity of 1800 μmol m^−2^ s^−1^ and constant temperature (25°C), with stepwise increase of external CO_2_ concentration (9 points ranging from 50 to 2000 μmol mol^−1^ with 2–4 min of adaptation). From the *A_n_*/*C_i_* curves, the maximal rate of Rubisco carboxylation (*V_cmax_*) and the rate of electron transport (*J*) were parametrized using the Farquhar model [37], with equations and constants from [38]. The light-saturated rate of electron transport driving regeneration of ribulose bisphosphate (*J_max_*) was calculated from *J* with equation and constants from [39]. The proportion of the variance explained by the model, *i.e.*, the model efficiency (ME) was calculated to confirm that the model could explain the measured data.

### Membrane-lipid analyses

A total lipid extract was obtained from 40–60 mg of frozen leaf tissue according to [40], diluted in 100 μl of methanol and subjected to liquid chromatography and mass spectrometry with MRMs as described in [41] using the head group specific fragments or neutral losses according to [42]. Neutral loss of 179, 341, 189, 141, 260 and 285 in positive mode was used for monogalactosyldiacylglycerol (MGDG), digalactosyldiacylglycerol (DGDG), phosphatidylglycerol (PG), phosphatidylethanolamine (PE), phosphatidylserine (PS) and phosphatidylinositol (PI), respectively. Precursors of 225 in negative mode were used for detecting sulfoquinovosyldiacylglycerol (SQDG), and positive mode precursors of 184 for phosphatidylcholine (PC). The content of individual glycolipids (MGDG, DGDG and SQDG) and phospholipids (PG, PE, PS, PI and PC) was expressed per leaf fresh weight and also used to calculate the molar glycolipid/phospholipid ratio.

### Statistical analyses

Four plants per treatment and time point were used for shoot analyses. Four-to-seven plants per treatment and time point were used for leaf analyses. Statistical analyses were performed using the software GraphPad InStat (GraphPad Software Inc., La Jolla, USA). Different treatments were compared with 1-way ANOVA. Where significant differences were found, the ANOVA was followed by Student-Newman-Keuls as posterior test. Data related to anatomical and ultrastructural parameters did not follow normal distribution. Therefore, the Kruskal-Wallis non-parametric ANOVA was used followed by the Mann–Whitney rank-sum test when appropriate. For all data, significant differences are described in the text at *P*<0.05. Multivariance analysis and clustering with principal component analysis (PCA), were done using the software PAST [43].

## Results

### Effect of the AM symbiosis on shoot growth and development

To test the effect of mycorrhization and P_i_ fertilization on shoot growth in our experimental conditions, dry weight (DW), P, N and C content were measured for shoots of plants at 2, 3, 4, 5, 6 and 8 wpi (Experiment 1). The intensity of mycorrhizal colonization in the roots (*M*%) and the arbuscules abundance (*A*%) were also estimated. Although the values for the *M* parameter in AM roots were high at all time points, the *A* parameter varied having the highest and lowest values at 3 and 8 wpi, respectively (Table 1). Roots from control and P_i_-fertilized plants did not display any fungal colonization at any time point (data not shown). Higher DW, P, N and C content were obtained for shoots of mycorrhized- than for control plants at all time points, but significantly higher values were obtained only at 3 wpi (S1 Table). Plants fertilized with P_i_ displayed similar shoot DW to the AM plants at 3 wpi, and significantly higher values were obtained for this parameter at later time points. Under P_i_ fertilization, shoots displayed significantly higher total nutrient content than the other treatments throughout the growth period.

Next, we tested the effect of mycorrhization and P_i_ fertilization on shoot development in our experimental conditions (Experiment 1) by measuring the number of metamers, number and length of primary branches, number of appeared metamers for MA and each primary branch over the period of 8 wpi (S2 Table). As in the case of the nutrient content, the number of metamers and primary branches at 3 wpi were significantly higher for AM- than for control plants. The P_i_-fertilized plants also developed significantly more metamers and branches than the control plants, and at 5–8 wpi significantly more even than the AM plants. At all time points, both the AM- and the P_i_-fertilized plants had longer primary branches than the control plants (S2 Table). Interestingly, at 3 wpi, the AM plants had significantly longer primary branches than the P_i_-fertilized plants.

**Figure 1:**
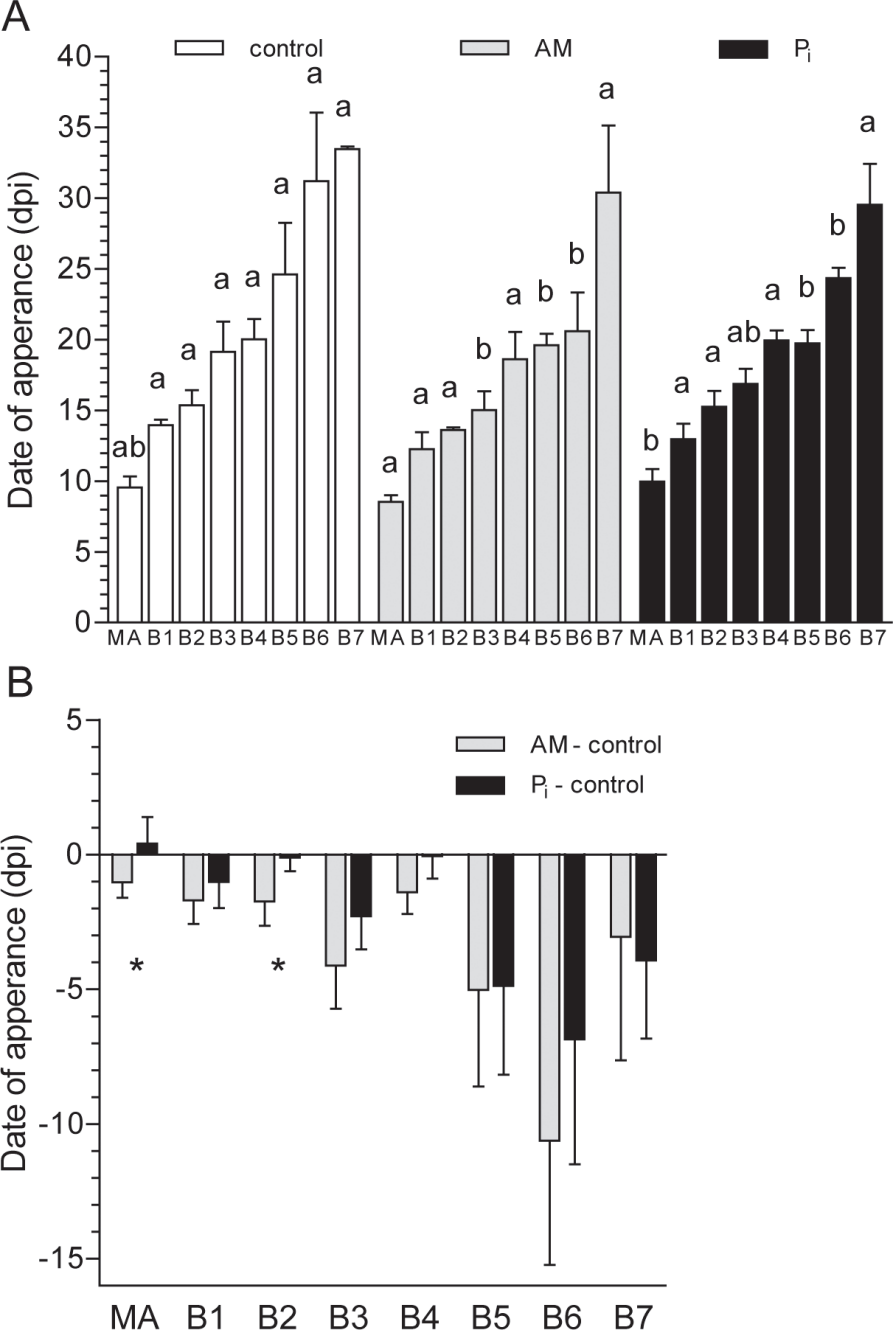
Date of growth axis appearance for control, mycorrhized (AM) and phosphate-fertilized plants (P_i_). (A) The date when each growth axis appeared was calculated as the x-intercept from linearized plots of those shown in S1 Fig. Values with different letters for each axis are significantly different across treatments according to one-way ANOVA followed by Student-Newman-Keuls test (*P*<0.05). (B) Difference in the date of growth axis appearance. The values obtained in (A) for each axis of the control plants were subtracted from the corresponding values obtained for the AM- and Pi-fertilized plants. *, Significantly different from each other. dpi, days post inoculation. For mycorrhization conditions, see Table 1.

These observations led us to hypothesize that mycorrhization stimulates the appearance of primary branches. To test this, the number of appeared metamers was recorded and plotted for each growth axis (*i.e.*, MA and B1-B7) over the period of 8 wpi (S1 Fig.). The initial rate of metamer appearance (*R_M_*) did not differ between the control- and the AM plants, but was significantly higher in the P_i_-treated plants (S1 Fig.). To calculate the date of axis appearance (counted as dpi), the curves for all growth axes except for MA could be fitted using a power equation (R^2^ ≥ 0.9). Distinct patterns for the date of appearance of each axis were observed. MA and B1 to B3 appeared stepwise in all treatments (Fig. 1A). Other branches appeared simultaneously within each treatment, namely B3 and B4 on the control plants, B4 to B6 on the AM plants, and B4 and B5 on the P_i_-fertilized plants. When comparing the date of appearance for each axis between treatments, statistically significant differences were observed for AM- versus control plants in the case of branches B3, B5 and B6, and for P_i_ versus control plants in the case of B5 and B6 (Fig. 1A). The non-significance for the other branches is mostly due to the fact that the measurements have been performed on different plants, increasing sample heterogeneity. In Fig. 1B, we subtracted the values obtained for the control plants from those corresponding to the AM- and P_i_-fertilized plants. MA and branches B1 to B4 appeared earlier on the AM- than on the control and P_i_-treated plants, whereas branches B5-B7 appeared simultaneously under AM and P_i_ fertilization conditions. These results indicate that the AM symbiosis had a stronger influence on the appearance of the first growth axes than P_i_ fertilization.

**Figure 2:**
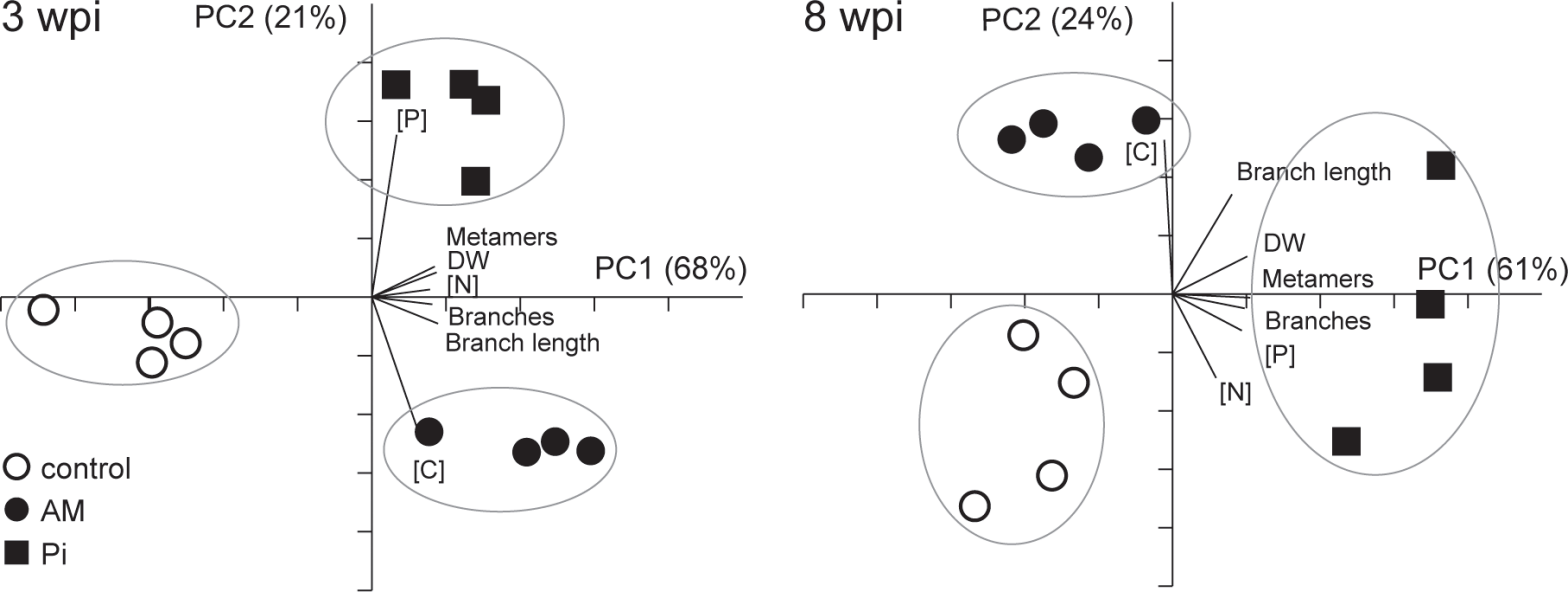
Principal component analysis score plot comparing the nutrient and developmental profile of control, mycorrhized (AM) and phosphate-fertilized plants (P_i_). The P, N and C concentrations ([P], [N] and [C]) of plants at 3 and 8 weeks *post* inoculation (wpi) were determined and expressed as percentage of shoot dry weight (DW). Each dot represents a single plant. The principal components (PC) explaining most of the variance in the data set are represented on the axis. Biplots indicate how each variable influences the distribution of the data-points. For mycorrhization conditions, see Table 1.

The various developmental and nutrient parameters provide distinct views of the influence of mycorrhization and P_i_ fertilization on shoot growth. To determine how these parameters are interrelated and the relationship with the extent of mycorrhizal colonization in the roots, PCA was carried out at 3 and 8 wpi. Principal component 1 (PC1) described >60% and PC2 about 20% of the variance at both selected time points (Fig. 2). This analysis did not position the AM treatment in between the control and P_i_ treatment, which would be expected if AM closely mimics P_i_ conditions. Instead, AM appeared as a clearly separated group from both control and P_i_ treatment. The P_i_-fertilized plants were separated from the non-fertilized (control and AM) plants mainly based on the P concentration (expressed as a percentage of shoot DW), whereas the C concentration and branch length separated the AM- from the non-mycorrhized (control and P_i_) plants. Shoot DW, number of metamers, length and number of primary branches closely correlated with the N concentration at 3 wpi. No such close correlation was observed at 8 wpi between the developmental parameters and any specific nutrient parameter. Taken together, our data indicate that P limits shoot growth in AM- and control plants, whereas the P demand of P_i_-fertilized plants appears to be satisfied. Increased P uptake by either fertilization or mycorrhization (at 3 wpi) is accompanied by an increased uptake of N and assimilation of C, leading to increased growth. A second limiting factor must exist in P_i_-fertilized plants, because P contents were considerably higher, whereas the other nutrient and developmental parameters displayed only a modest increase when compared to AM plants. This could reside in the genetic background of *M. truncatula*, which does not allow plants to develop and grow proportionally to the available P in their leaves. The observed increase in growth of P_i_-fertilized and AM plants points to an increased photosynthetic production. This could be achieved either (1) by an increase in the surface for sunlight absorption and/or (2) by an increase in the photosynthetic activity per leaf area.

### Effect of the AM symbiosis on photosynthetic tissue

According to the first hypothesis, an increase in photosynthetic production by AM would be achieved by enhancing the surface to harvest sunlight. To test whether AM affects leaf size and structure, we performed an additional experiment (Experiment 2), and selected two time points, namely 3 and 5 wpi, corresponding to distinct arbuscules abundances in the AM roots (Table 2). The leaflet area and thickness were significantly larger for the AM- than for the control plants at both time points (Table 2). Under P_i_-fertilization the leaflet area at 5 wpi increased more than under AM conditions, whereas the thickness was intermediate between control and AM. Representative semi-thin sections of control and AM leaflets taken at 3 wpi are shown in S2A Fig. Neither the thickness of the palisade or the spongy parenchyma cell layers nor the ratio between them significantly differed among the treatments. The number of cells in the 100-μm leaflet sections did not change significantly between treatments (Table 2), indicating that, additionally, the width of the cells was invariable with the treatments. From this we can conclude that the increase in the leaflet area is the result of an increase in cell number per leaflet rather than in cell size. The number of chloroplasts in the palisade cell sections was significantly higher for the AM- as compared to the control leaflets both at 3 and 5 wpi (Table 2). The spongy cell sections had significantly higher number of chloroplasts in the AM- than in the control leaflets at 3 wpi, but no significant differences were observed at 5 wpi. The number of chloroplasts per cell sections in P_i_-leaflets were intermediate between control and AM at 3 wpi, and were similar to control at 5 wpi.

Chloroplast morphology of the different treatments at 3 and 5 wpi was studied on ultrathin sections with transmission electron microscopy. Sectional images of chloroplasts were in general slightly longer and thinner in the palisade parenchyma than in spongy parenchyma cells (S2B, C Fig.). The size (length and width) and shape (represented as length/width ratio) of palisade and spongy parenchyma chloroplasts were quantified (Table 3). Interestingly, the chloroplasts from AM palisade parenchyma had a slightly more flattened shape at 3 wpi, *i.e.*, their section was longer and significantly narrower than that of the other plants at the same wpi. Spongy parenchyma chloroplasts were in general less elongated than those in the palisade parenchyma. The length of spongy parenchyma chloroplasts did not show any statistical differences due to treatments. At 3 wpi, the AM spongy parenchyma chloroplasts were significantly narrower than the control ones resulting in significantly higher length/width ratio, but these differences were less obvious in chloroplasts at 5 wpi. For comparison, a modified shape of the chloroplasts (narrower) was observed at 5 wpi in samples from P_i_-fertilized plants, resulting in a similar length/width ratio as in the AM samples. No visual differences were found in the chloroplast ultrastructure, *i.e.*, organization of thylakoid membranes among the different treatments. The number and size of starch grains greatly varied among samples and leaves. Taken together, these results indicate that both mycorrhization and P_i_ fertilization modified the structure of the photosynthetically active tissue, most likely to increase the surface for sunlight absorption. In addition, AM increased the number of chloroplasts found in a cell section.

**Table 3:** Dimensions of leaf chloroplasts from control, mycorrhized (AM) and phosphate-fertilized plants (P_i_).

**Parameter**	**wpi**	**Control**	**AM**	**P_i_**
Palisade chloroplast length (μm)	3	6.5 ± 1.6^a^	6.8 ± 1.9^a^	6.6 ± 1.5^a^
	5	6.0± 1.6^a^	6.3 ± 2.2^a^	6.9 ± 1.6^b^
Palisade chloroplast width (μm)	3	2.4 ± 0.9^a^	2.2 ± 1.5^c^	2.8 ± 1.0^b^
	5	2.4 ± 0.9^a^	2.2 ± 0.9^b^	2.2 ± 0.9^b^
Palisade chloroplast length/width	3	3.0 ± 1.3^a^	3.5 ± 1.5^b^	2.6 ± 1.0^a^
	5	2.8 ± 1.1^a^	3.4 ± 1.7^b^	3.6 ± 1.4^b^
Spongy chloroplast length (μm)	3	5.7 ± 1.4^a^	5.9 ± 2.1^a^	5.7 ± 1.4^a^
	5	5.4 ± 1.3^a^	5.4 ± 1.3^a^	5.7 ± 1.5^a^
Spongy chloroplast width (μm)	3	2.4 ± 0.9^a^	2.1 ± 0.9^b^	2.4 ± 0.8^a^
	5	2.5 ± 0.9^a^	2.3 ± 0.9^a^	2.1 ± 0.8^b^
Spongy chloroplast length/width	3	2.6 ± 0.9^a^	3.0 ± 1.3^b^	2.5 ± 0.9^a^
	5	2.4 ± 1.0^a^	2.8 ± 1.2^b^	3.0 ± 1.3^b^

The data are means of four plants ± SD. Values with different letters in each row are significantly different across treatments according to Kruskal-Wallis non-parameteric ANOVA followed by Mann-Whitney rank-sum test (*P*<0.05). For mycorrhization conditions, see Table 2 footnote. wpi, weeks *post* inoculation.

### Effect of the AM symbiosis on leaf photosynthesis

According to the second hypothesis, the increase in photosynthetic production would rely on an increase in the specific photosynthetic activity (per unit leaf area). To test this, the photosynthetic CO_2_ fixation was studied in Experiment 2 by recording *A_n_/C_i_* curves on leaflets (S3 Fig.). No significant difference in *A_n_* expressed per area was observed among the treatments at 3 and 5 wpi at any given *C_i_*, including the ambient CO_2_ concentration (*A_ambient_*) (Table 4). No difference among the treatments was observed for *V_cmax_* or *J_max_* when expressed per area, with the exception of *V_cmax_* at 3 wpi, which was significantly lower in the AM- compared to the control leaflets. The whole leaflet *A_ambient_, V_cmax_* and *J_max_* were significantly higher for AM at both 3 and 5 wpi (Table 4) due to the larger area compared to the control leaflets. The same parameters were also found significantly higher under P_i_ fertilization at both time points, and in the case of *J_max_*, even higher than for the AM leaflets. Despite the observed increase in chloroplast number per cell section, no significant difference was found between treatments in regard to carotenoids, total Chl *a* and *b* content per leaf area, or in the Chl *a/b* ratio (S3 Table). Chlorophyll fluorescence measurements indicated similar photochemistry based on no significant differences in the *F_v_/F_m_* ratio, which was on average 0.8 for all treatments, *i.e.*, typical value for healthy plants [36]. Taken together, our data indicate that AM symbiosis did not alter photosynthetic activity per leaf area. Thus, an increase in photosynthetic production was achieved due to an increased surface for sunlight absorption.

**Table 4:** The photosynthetic parameters of leaves from control, mycorrhized (AM) and phosphate-fertilized plants (P_i_).

**Parameter**	**wpi**	**Per area (m^−2^)**			**Per leaflet**		
		**Control**	**AM**	**P_i_**	**Control**	**AM**	**P_i_**
*A_ambient_*(μmol CO_2_ s^−1^)	3	33.0 ± 0.8^a^	31.9 ± 1.2^a^	32.0 ± 0.6^a^	45.1 ± 8.5^a^	66.0 ± 6.5^b^	73.0 ± 9.7^b^
	5	33.2 ± 3.5^a^	31.5 ± 3.1^a^	30.8 ± 3.6^a^	55.6 ± 4.1^a^	61.6 ± 8.1^b^	84.9 ± 13.1^b^
*V_cmax_*(μmol CO_2_ s^−1^)	3	145 ± 6^a^	132 ± 6^b^	133 ± 6^b^	189.9 ± 1.2^a^	273.2 ± 1.2^b^	303.2 ± 1.8^b^
	5	142 ± 13^a^	132 ± 11^a^	127 ± 14^a^	230.0 ± 2.0^a^	257.4 ± 1.5^b^	354.3 ± 7.1^c^
*J_max_*(μmol e^−^ s^−1^)	3	221 ± 6^a^	198 ± 21^a^	199 ± 15^a^	289.5 ± 1.2^a^	409.8 ± 4.6^b^	453.7 ± 0.4^c^
	5	220 ± 6^a^	203 ± 21^a^	216 ± 14^a^	356.4 ± 9.7^a^	395.8 ± 2.9^b^	602.6 ± 7.4^c^

*A_ambient_* is photosynthetic CO_2_ fixation at ambient CO_2_ concentration. The maximal rate of Rubisco carboxylation (*V_cmax_*) and electron transport (*J_max_*), were modeled from an *A_n_/C*_i_ curve (S3 Fig.) measured at saturating light (1800 μmol photons m^−2^ s^−1^). Model efficiency 0.99. The data are means of six plants ± SD. Values with different letters for each parameter in each row are significantly different among treatments according to one-way ANOVA followed by Student-Newman-Keuls test (*P*<0.05); For mycorrhization conditions, see Table 2 footnote. wpi, weeks *post* inoculation.

### Effect of the AM symbiosis on leaf phospholipid content

**Figure 3:**
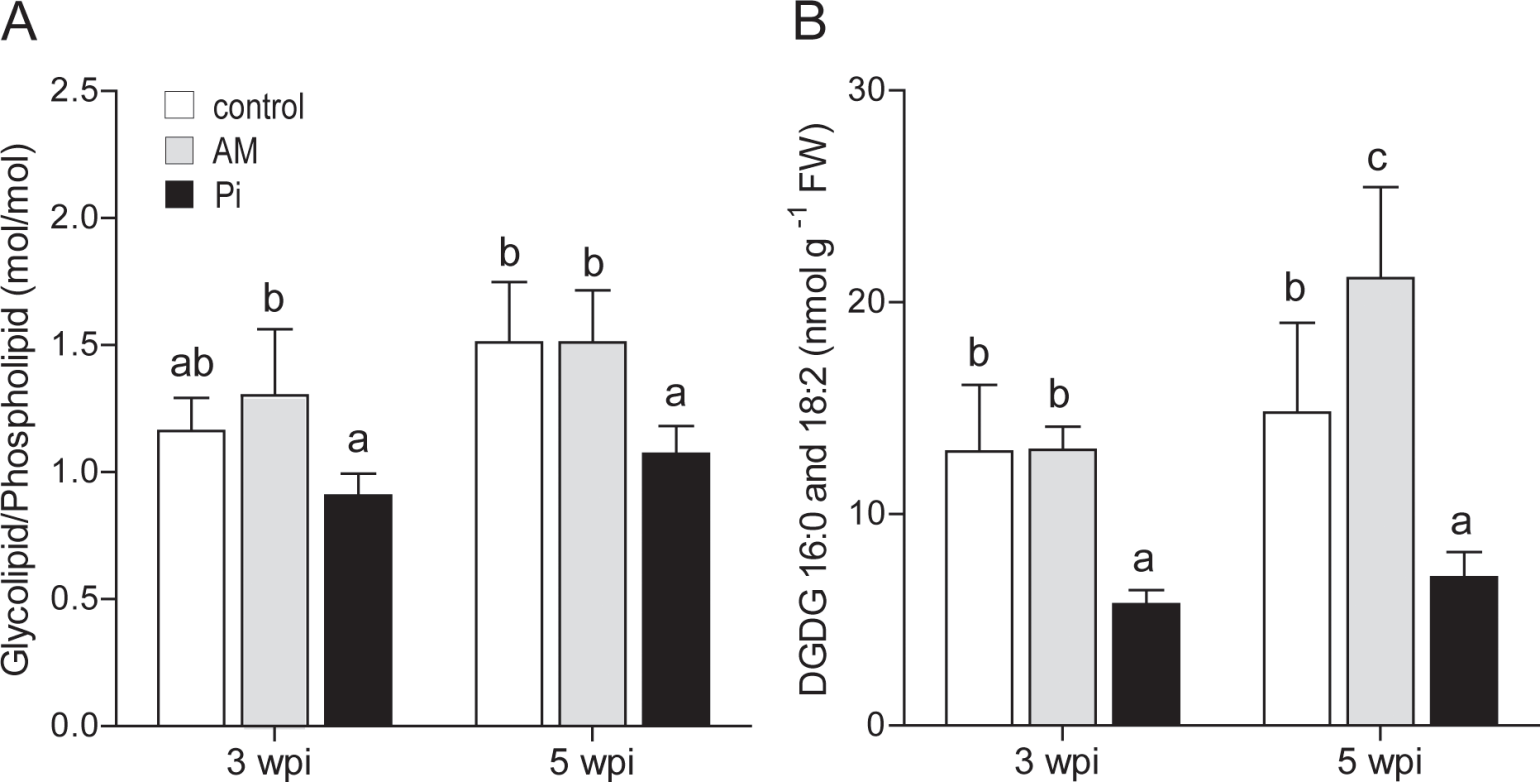
Membrane-lipid content of leaves from control, mycorrhized (AM) and phosphate-fertilized plants (P_i_). (A) Leaves were harvested from plants at 3 and 5 weeks *post* inoculation (wpi), the membrane lipids were extracted and analyzed by liquid chromatography and mass spectrometry. Molar ratio of glycolipids to phospholipids was calculated based on data for individual lipid species presented in S4 Fig. (B) Content of 16:0- and 18:2 digalactosyldiacylglycerol (DGDG) lipid species is expressed per leaf fresh weight (FW). For mycorrhization conditions, see Table 2 footnote. The data are means of six plants ± SD. Values with different letters are significantly different across treatments according to one-way ANOVA followed by Student-Newman-Keuls test (*P*<0.05).

To examine if the AM symbiosis influences the leaf thylakoid or extraplastidic membrane-lipid composition, glycerolipids were extracted at 3 and 5 wpi and quantified (Experiment 2). The glycerolipid profile at 3 wpi did not significantly differ between the treatments, and was dominated by MGDG, followed by DGDG, PG, PC and PE, SQDG and finally smaller proportions of PI and PS (S4A Fig.). However, there was a shift towards significantly more phospholipids resulting in a lower glycolipid/ phospholipid ratio in leaves of the P_i_-fertilized plants (Fig. 3A). At 5 wpi, significantly more glycolipids were found in the leaves of AM- than in the other treatments (S4 Fig.). In addition, the AM leaves contained significantly higher amounts of the phospholipids PC and PE than the control leaves, but similarly high as the P_i_ leaves. However, as at 3 wpi, the glycolipid/phospholipid ratio was significantly lower in the P_i_-fertilized plants, but not between the mycorrhized and the control plants (Fig. 3A).

Next, we investigated the levels of the DGDG species containing 16:0 and 18:2 fatty acids, known to replace phospholipids during P_i_ starvation [44]. The addition of exogenous P_i_ to the plants at 3 wpi caused about 3-fold reduction in the leaf content of these lipid species when compared to the control and AM-conditions (Fig. 3B). At 5 wpi, the content of these species was found 3- and 4-fold higher in leaves of the control and AM plants, respectively, than in P_i_ –fertilized plants. These results are in line with the overall lower P availability for control- and AM- as compared to the P_i_-treated plants.

## Discussion

The current development of agriculture is partly driven by the implementation of new practices, including co-culturing, reduction of the field inputs (fertilizers, pesticides, fungicides) and the use of bio-effectors such as AM. Despite the availability of much data on the changes induced by these new practices in plants, the mechanisms involved remained elusive. Therefore, additional data are needed to optimize the development of the new agricultural practices. This study is dedicated to the elucidation of mechanisms triggered by mycorrhization and allowing the improvement of shoot growth and development of the model legume *M. truncatula*. This plant was chosen because legumes are the second most important source of human food and animal forage, providing grains enriched in proteins.

We have conducted measurements on *M. truncatula* shoots in two experiments: nutrient content, shoot growth and development (Experiment 1), leaf anatomy and photosynthesis (Experiment 2). The intensity of colonization and arbuscules abundance varied over time and between experiments (Tables 1 and 2). Table 5 summarizes the observed alterations in AM- versus control and P_i_-fertilized versus control plants for the time point where the arbuscules abundance was similarly high in the two experiments (50–60%), *i.e*., 3 wpi for Experiment 1 and 5 wpi for Experiment 2.

**Table 5:** Summary of alterations observed in mycorrhized (AM) and phosphate-fertilized (P_i_) as compared to control plants.

**Parameter**	**AM**	**P_i_**	**Experiment**
**Shoot nutrient**			
P content	+	++	1
N content	+	+	1
C content	+	+	1
**Shoot growth and development**			
Dry weight	+	+	1
Number of metamers and branches	+	+	1
Branch length	++	**+**	1
First growth axes appearance	+	No	1
Late growth axes appearance	+	+	1
**Leaf anatomy**			
Leaflet area	+	+	2
Leaflet thickness	+	+	2
Number of chloroplasts per cell section	+	No	2
Chloroplast length/width	+	+	2
**Leaf photosynthesis**			
Electron transport rate per leaflet	+	++	2
Net CO_2_ assimilation per leaflet	+	+	2

The summarized alterations are for the time points with similarly high arbuscules abundance in Experiment 1 (3 wpi) and Experiment 2 (5 wpi). ‘+’ indicates a stimulatory effect, whereas ‘No’ indicates no effect of the respective treatment as compared to control plants. It is of note that a similar pattern of alterations was observed at 3 wpi in Experiment 2. For mycorrhization conditions, see Tables 1 and 2.

### AM symbiosis increases surface for better sunlight absorption

In Experiment 1, we show that the shoots of mycorrhized *M. truncatula* produced branches earlier than the control plants (Fig. 1B). This result is of great importance because it provides clues as to why mycorrhized plants produced more and longer shoot branches than the control ones at the same time after inoculation. The comparison of the date of growth axis appearance on the AM-treated and P_i_-fertilized versus the control plants suggests a specific mycorrhization effect for the first growth axes (MA, B1 to B4) since for the late (B5 to B7) branches, both treatments had equivalent date of appearance.

Significant stimulation in growth and development by the AM treatment as compared to the control was observed at 3 wpi in Experiment 1 (S1 and S2 Tables). The finding that the AM plants develop more and longer branches indicates that their shoots occupy a larger surface than the control shoots. For instance, Zhang et al. [45] reported similar data, *i.e*., higher canopy radius/biomass in *M. sativa* (alfalfa) plants grown in AM soil as compared to plants grown in fungicide-treated (non-mycorrhized) soil. The growth stimulation can be explained by the increase of P and N content found in AM plants [46–49]. In this study, the content of N, P and C was also enhanced at 3 wpi in the shoots of AM- when compared to the control plants (S1 Table). The increased growth, development and nutrient uptake at 3 wpi in the AM- versus control plants (Fig. 2) point to an improved photosynthetic activity by mycorrhization. One possibility to achieve this is by increasing the photosynthetic activity per leaf area. This seems very unlikely in our study, since CO_2_ assimilation per area was not altered either by mycorrhization or P_i_ fertilization (Table 4). Thus, the increase in the canopy size is the process by which *M. truncatula* increases the photosynthetic production required to sustain the additional (fungal) sinks. This is indeed supported by the finding that the AM plants have larger and thicker leaves containing more chloroplasts per cell section as compared to the control plants (Table 2). Similarly, the number of chloroplasts and also leaf thickness increased in the leaves of mycorrhized *Eleusine coracana* (finger millet) [50]. Interestingly, several reports indicated that AM symbiosis increased the number of plastids in root cells developing arbuscules [51,52], suggesting that plastid multiplication is a general phenomenon triggered by mycorrhization. This is in line with our observations that chloroplast number did not increase in P_i_-fertilized versus control plants. The fact that the photosynthetic activity per area did not differ between AM- and P_i_-fertilized plants indicates that the changes in chloroplast number in the AM plants did not impact the photosynthetic capacity. The observation of increased leaf area upon mycorrhization in our study is also in line with several previous reports for *M. truncatula* [21,22] and *Prunus armeniaca* [53]. Nevertheless, the leaf area was unaffected by AM colonization in the case of *Lolium perenne* [54]. Another strategy to increase the sun exposure is by growing taller, as found in mycorrhized trees such as *Olea europea* (olive) [55], *Sophora davidii* (shrub pagoda tree) [56] and *Prunus armeniaca* (apricot) [53].

When comparing the AM effects with those of P_i_ fertilization, in this study we could distinguish the specific influence of AM symbiosis on plant physiological characteristics. The AM-specific effects were the earlier appearance of the first growth axis on the plants and the altered chloroplast number per cell section (Table 5). All other changes were also induced by P_i_ fertilization, indicating that they could be the result of an increased P_i_ supply in the leaves. The shared changes by AM and P_i_ treatments are in line with previous observations by Bucciarelli et al. [19] for *M. truncatula* grown under P_i_-deprived and P_i_ optimal conditions. The various processes affected by mycorrhization may have distinct time scales and modes of regulation, since alterations in the shoot were observed only at the age corresponding to the highest arbuscules abundance, whereas those at leaf and subcellular level were also evident at low arbuscules abundance.

### AM symbiosis stimulates total leaf photosynthesis without altering activity per area

Lower total P contents were determined in the shoots of control and AM- as compared to the P_i_-fertilized plants (S1 Table). The significant increase in the glycolipid /phospholipid ratio and in the abundance of the 16.0- and 18:2-DGDG lipid species in the control and AM leaves (Fig. 3) supports the notion of a lower availability of P in these treatments. However, the observation of a significantly higher *V_cmax_* per leaf area in the control as compared to the AM- and P_i_-fertilized plants at 3 wpi (Table 4) indicates that in our experimental conditions the (low) P_i_ levels did not limit photosynthesis. At 5wpi, photosynthetic parameters (A*_ambient_*, V*_cmax_* and *J_max_*) when expressed per leaf area were not altered either by mycorrhization or P_i_ fertilization. Since these both conditions modified the photosynthetic tissue, resulting in about two-fold larger leaflet area, an increased whole leaflet photosynthetic capacity could be estimated (Table 5). Based on these results, we suggest that the carbon cost of the AM symbiosis was more than compensated for by the increased CO_2_ assimilation, resulting in a net increase in shoot DW, which was found correlated with its C concentration (Fig. 2). Differences in the maximum quantum yield of photosystem II (estimated by *F_v_/F_m_*) and pigment content in leaves were not significant between the control and the AM treatment, supporting earlier observations for *Plantago lanceolata* [17]. The discrepancy between our results and some of those published previously (decrease: [12], increase: [13]) could be due to the type of inoculum used and plant species analysed.

## Conclusions

The results presented here show that mycorrhization in *M. truncatula* influences the development of the photosynthetically active tissue in addition to the well-documented effects on plant growth and shoot branching. The effects are either unique to AM or shared with P_i_ fertilization (Table 5), and result in increased surface for sunlight capture and better photosynthetic production. The observed alterations in the photosynthetic tissue are intriguing and worth further investigation using large-scale approaches, such as transcriptomics and metabolomics.

## Supporting Information

S1 Fig Changes over time in the number of appeared metamers on main axis (MA) and on branches B1 to B7 from control, mycorrhized (AM) and phosphate-fertilized (P_i_) plants. The total number of metamers was plotted for each growth axis (panels MA and B1 to B7) as a function of age expressed as days *post* inoculation (dpi). In the last panel, the initial rate of metamer appearance on each branch (*R_M_*) was calculated from linear regression of all time points of each curve. For mycorrhization conditions, see Table 1. The data are means of four plants ± SD. Values with different letters in the last panel are significantly different across treatments according to one-way ANOVA followed by Student-Newman-Keuls test (*P*<0.05).
(TIF)

S2 Fig Leaf and chloroplast structure of control (left panels) and mycorrhized (AM, right panels) plants at three weeks *post* inoculation (wpi). (A) Representative light micrographs of semi-thin sections showing increased leaf thickness for the AM- as compared to the control plants. Scale bar: 100 μm. Representative transmission electron micrographs of chloroplasts from palisade (B) and spongy (C) parenchyma cells displaying slightly more elongated and narrow shape in the AM- as compared to the control leaves. Scale bar: 1 μm. For mycorrhization conditions, see Table 2 footnote.
(TIF)

S3 Fig Photosynthetic assimilation of CO_2_ (*A_n_*) of control, mycorrhized (AM) and phosphate-fertilized (P_i_) plants plotted against intracellular CO_2_ concentration (*C_i_*). Assimilation at ambient CO_2_ concentration is indicated with an arrow. For mycorrhization conditions, see Table 2 footnote. The data are means of six plants ± SD. wpi, weeks *post* inoculation.
(TIF)

S4 Fig Membrane lipid composition of control, mycorrhized (AM) and phosphate-fertilized (P_i_) plants. The lipids were extracted from fresh leaves of plants at 3 and 5 weeks *post* inoculation (wpi), and the various species of glycolipids and phospholipids were quantified with liquid chromatography and mass spectrometry. For mycorrhization conditions, see Table 2 footnote. MGDG, monogalactosyldiacylglycerol; PC, phosphatidylcholine; PE, phosphatidylethanolamine; PG, phosphatidylglycerol; PI, phosphatidylinositol; PS, phosphatidylserine; SQDG, sulfoquinovosyldiacylglycerol. The data are expressed per leaf fresh weight (FW) and are means of seven plants ± SD. Values with different letters are significantly different across treatments according to one-way ANOVA followed by Student-Newman-Keuls test (*P*<0.05).
(TIF)

S1 Table Shoot dry-weight (DW) and content of phosphorus (P), nitrogen (N) and carbon (C) of control, mycorrhized (AM) or phosphate-fertilized (P_i_) plants. For mycorrhization conditions, see Table 1. The data are means of four plants ± SD. Values with different letters in each row are significantly different across treatments according to one-way ANOVA followed by Student-Newman-Keuls test (*P*<0.05). wpi, weeks *post* inoculation.
(DOCX)

S2 Table Shoot developmental parameters of control, mycorrhized (AM) and phosphate-fertilized (P_i_) plants. For mycorrhization conditions, see Table 1. The data are means of four plants ± SD. Values with different letters in each row are significantly different across treatments according to one-way ANOVA followed by Student-Newman-Keuls test (*P*<0.05). wpi, weeks *post* inoculation.
(DOCX)

S3 Table Leaf chlorophyll and carotenoid content of control, mycorrhized (AM) or P_i_-fertilized plants. The pigments were extracted using methanol and quantified spectrophotometrically. The data are expressed per leaf area and are means of four plants ± SD. There is no significant difference across treatments (at *P*<0.05). For mycorrhization conditions, see Table 1. wpi, weeks *post* inoculation.
(DOCX)
