# Supplementary figures and images for: Mycorrhiza Symbiosis Increases the Surface for Sunlight Capture in Medicago truncatula for Better Photosynthetic Production
Source: PLoS One. 2015 Jan 23;10(1):e0115314. doi: 10.1371/journal.pone.0115314 (PMC4304716; doi:10.1371/journal.pone.0115314)

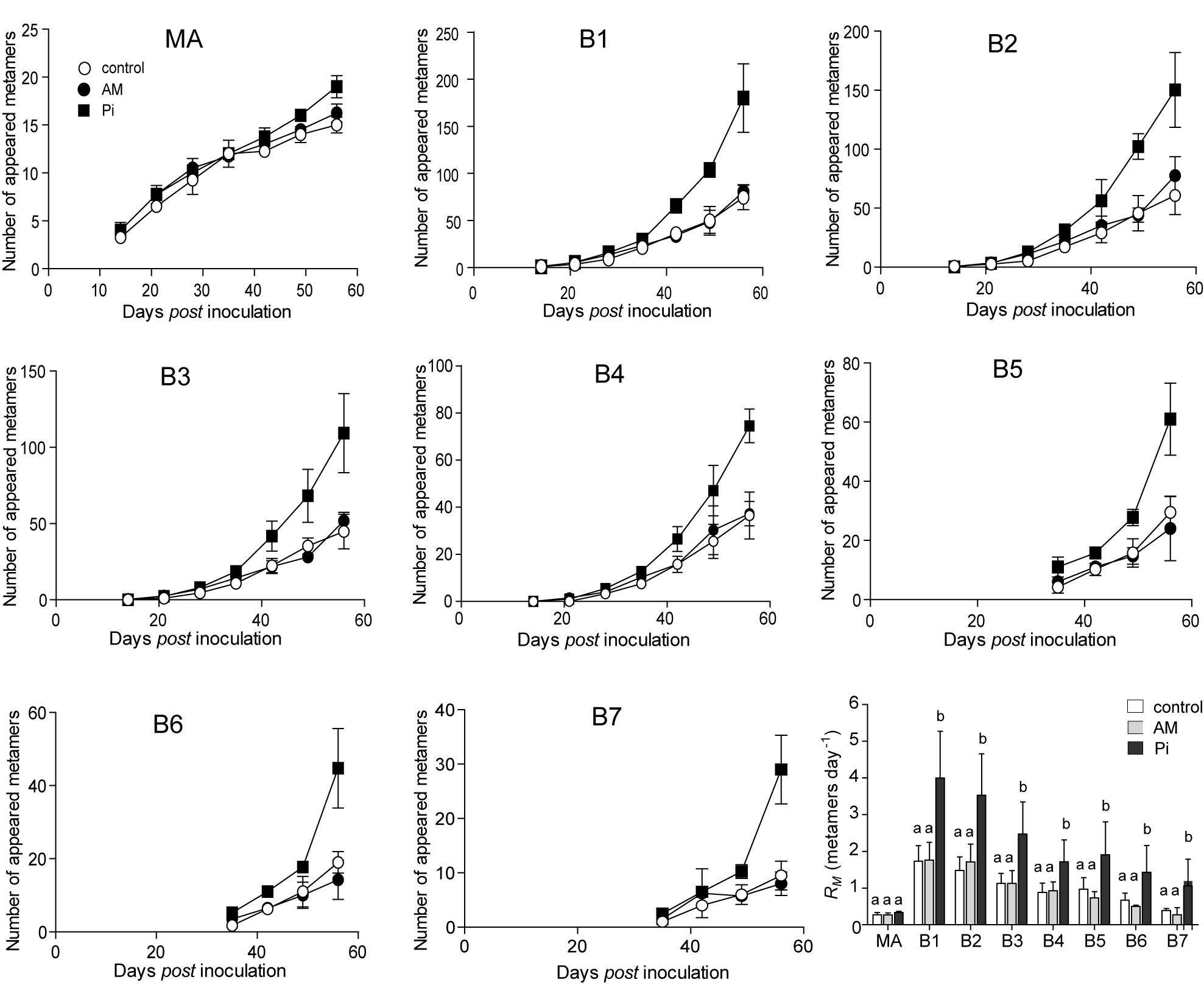

Supplement: S1 Fig — The total number of metamers was plotted for each growth axis (panels MA and B1 to B7) as a function of age expressed as days post inoculation (dpi). In the last panel, the initial rate of metamer appearance on each branch (RM) was calculated from linear regression of all time points of each curve. For mycorrhization conditions, see Table 1. The data are means of four plants ± SD. Values with different letters in the last panel are significantly different across treatments according to one-way ANOVA followed by Student-Newman-Keuls test (P<0.05). (TIF) [file pone.0115314.s001.tif]

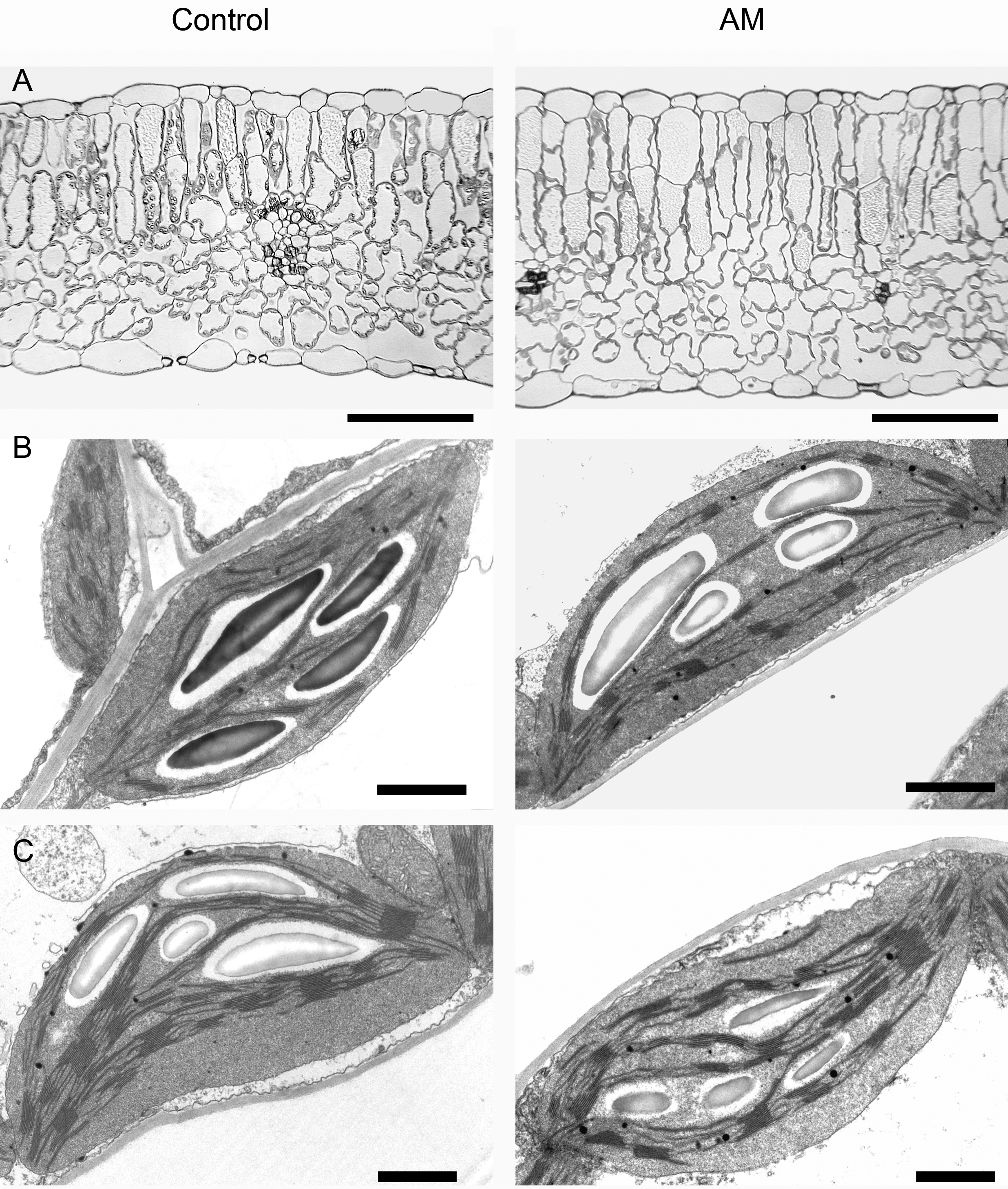

Supplement: S2 Fig — (A) Representative light micrographs of semi-thin sections showing increased leaf thickness for the AM- as compared to the control plants. Scale bar: 100 μm. Representative transmission electron micrographs of chloroplasts from palisade (B) and spongy (C) parenchyma cells displaying slightly more elongated and narrow shape in the AM- as compared to the control leaves. Scale bar: 1 μm. For mycorrhization conditions, see Table 2 footnote. (TIF) [file pone.0115314.s002.tif]

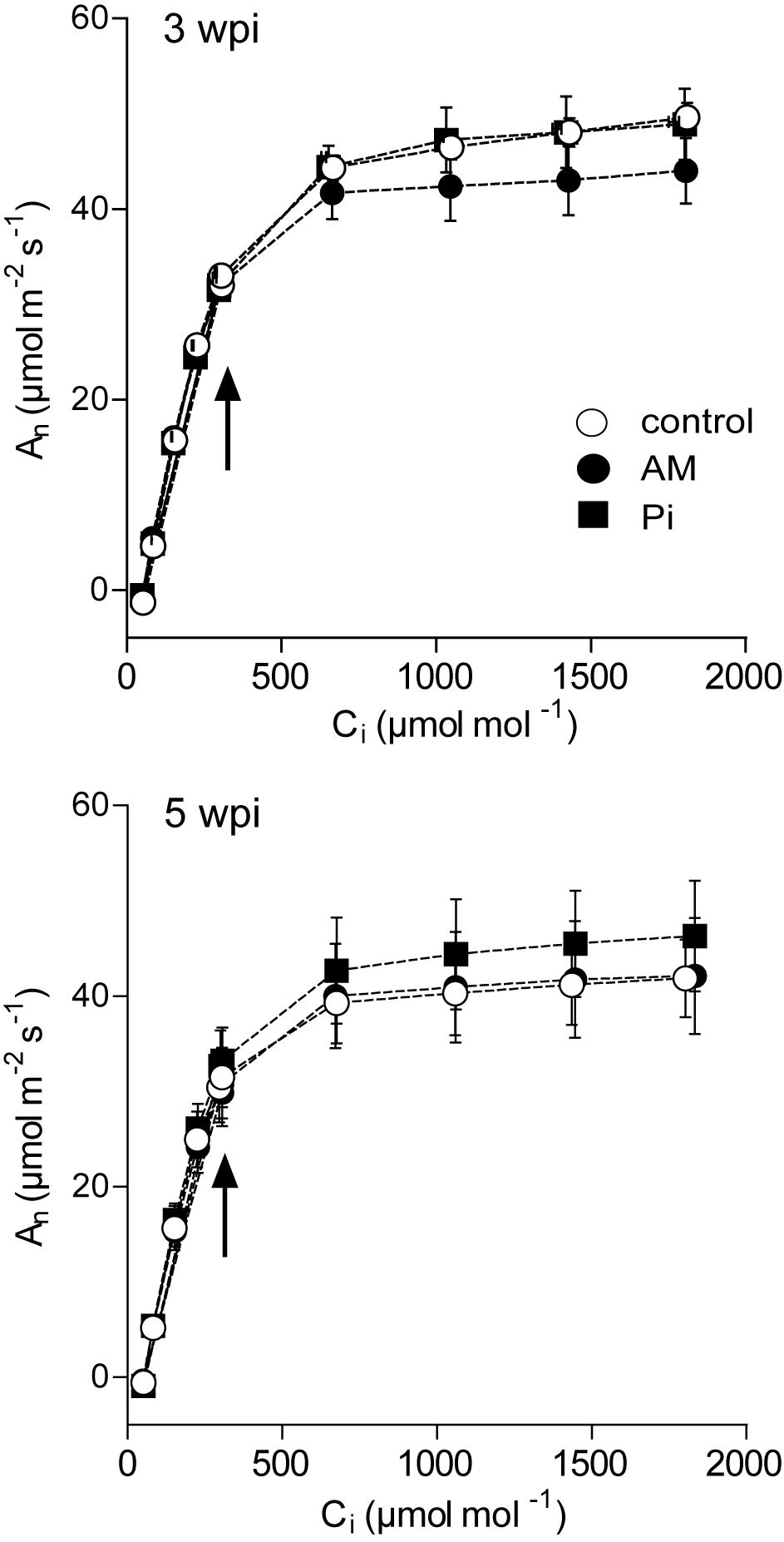

Supplement: S3 Fig — Assimilation at ambient CO2 concentration is indicated with an arrow. For mycorrhization conditions, see Table 2 footnote. The data are means of six plants ± SD. wpi, weeks post inoculation. (TIF) [file pone.0115314.s003.tif]

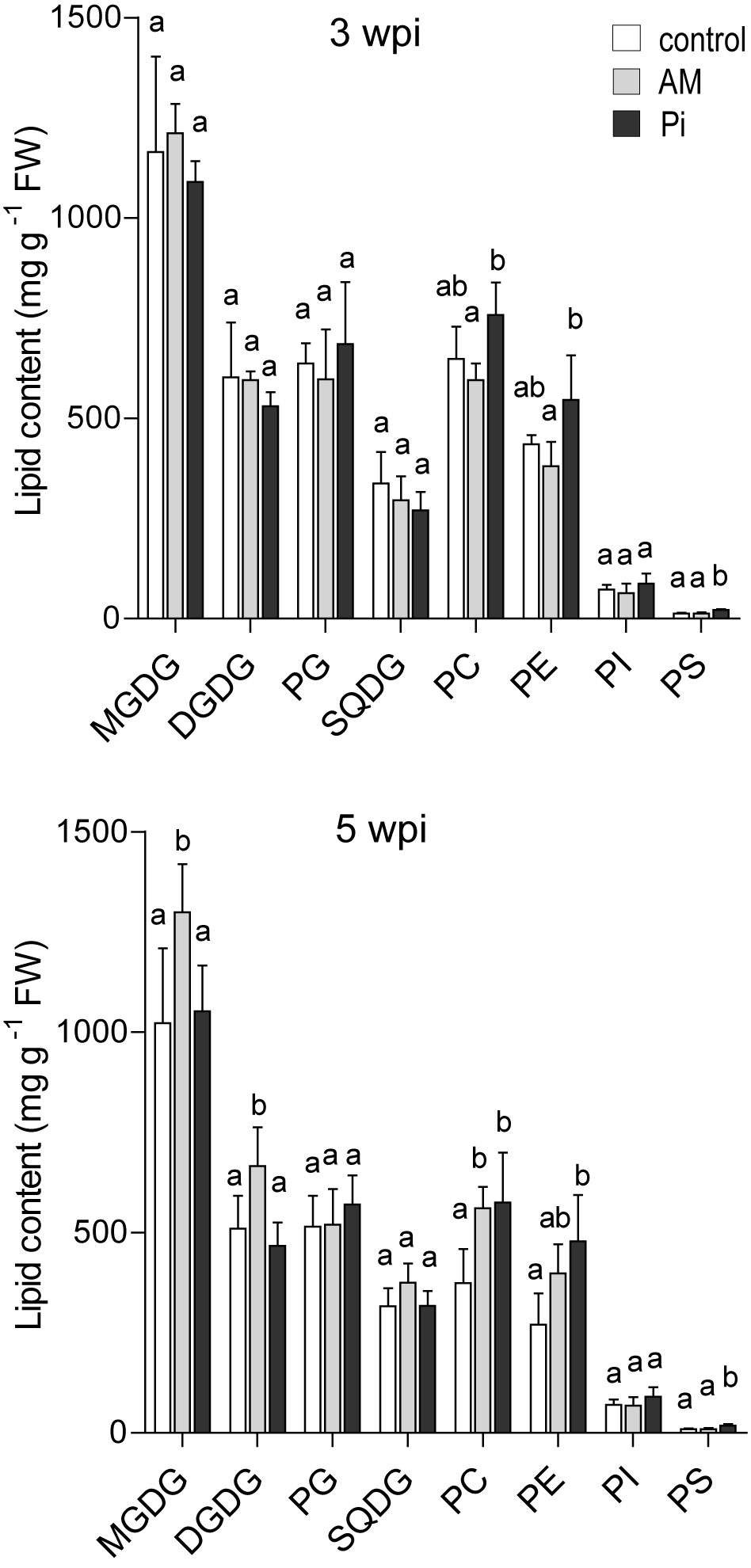

Supplement: S4 Fig — The lipids were extracted from fresh leaves of plants at 3 and 5 weeks post inoculation (wpi), and the various species of glycolipids and phospholipids were quantified with liquid chromatography and mass spectrometry. For mycorrhization conditions, see Table 2 footnote. MGDG, monogalactosyldiacylglycerol; PC, phosphatidylcholine; PE, phosphatidylethanolamine; PG, phosphatidylglycerol; PI, phosphatidylinositol; PS, phosphatidylserine; SQDG, sulfoquinovosyldiacylglycerol. The data are expressed per leaf fresh weight (FW) and are means of seven plants ± SD. Values with different letters are significantly different across treatments according to one-way ANOVA followed by Student-Newman-Keuls test (P<0.05). (TIF) [file pone.0115314.s004.tif]
