# Supplementary material for: Mycorrhiza Symbiosis Increases the Surface for Sunlight Capture in Medicago truncatula for Better Photosynthetic Production
Source: PLoS One. 2015 Jan 23;10(1):e0115314. doi: 10.1371/journal.pone.0115314 (PMC4304716; doi:10.1371/journal.pone.0115314)
Supplement: S1 Table — For mycorrhization conditions, see Table 1. The data are means of four plants ± SD. Values with different letters in each row are significantly different across treatments according to one-way ANOVA followed by Student-Newman-Keuls test (P<0.05). wpi, weeks post inoculation. (DOCX) [file pone.0115314.s005.docx]

**Table S1. Shoot dry-weight (DW) and content of phosphorus (P), nitrogen (N) and carbon (C) of control, mycorrhized (AM) or phosphate-fertilized (P_i_) plants*.***

| **Parameter** | **wpi**  **(wpi)** | **Control** | **AM** | **P_i_** |
| --- | --- | --- | --- | --- |
| DW  (g shoot^-1^) | 2 | 0.024 ± 0.002^a^ | 0.038 ± 0.012^a^ | 0.034 ± 0.010^a^ |
|  | 3 | 0.094 ± 0.017^a^ | 0.16 ± 0.02^b^ | 0.16 ± 0.02^b^ |
|  | 4 | 0.23 ± 0.04^a^ | 0.48 ± 0.10^ab^ | 0.71 ± 0.33^b^ |
|  | 5 | 0.61 ± 0.16^a^ | 0.91 ± 0.29^a^ | 1.3 ± 0.3^b^ |
|  | 6 | 1.4 ± 0.4^a^ | 1.7 ± 0.3^a^ | 2.9 ± 0.7^b^ |
|  | 8 | 3.6 ± 0.6^a^ | 5.0 ± 0.7^a^ | 8.8 ± 1.6^b^ |
| P content  (mg shoot^-1^) | 2 | 0.031 ± 0.003^a^ | 0.077 ± 0.020^a^ | 0.42 ± 0.18^b^ |
|  | 3 | 0.15 ± 0.04^a^ | 0.34 ± 0.06^b^ | 2.4 ± 0.2^c^ |
|  | 4 | 0.35 ± 0.05^a^ | 0.99 ± 0.25^a^ | 9.4 ± 4.4^b^ |
|  | 5 | 1.1 ± 0.3^a^ | 1.5 ± 0.3^a^ | 19 ± 6^b^ |
|  | 6 | 3.9 ± 2.0^a^ | 3.0 ± 0.3^a^ | 26 ± 5^b^ |
|  | 8 | 4.3 ± 1.1^a^ | 5.5 ± 0.8^a^ | 58 ± 12^b^ |
| N content  (mg shoot^-1^) | 2 | - | - | - |
|  | 3 | 4.5 ± 0.8^a^ | 9.1 ± 1.2^b^ | 8.9 ± 1.1^b^ |
|  | 4 | 11 ± 2^a^ | 27 ± 4^ab^ | 43 ± 21^b^ |
|  | 5 | 29 ± 8^a^ | 49 ± 13^a^ | 84 ± 17^b^ |
|  | 6 | 82 ± 25^a^ | 94 ± 19^a^ | 170 ± 30^b^ |
|  | 8 | 170 ± 40^a^ | 230 ± 40^a^ | 450 ± 90^b^ |
| C content  (g shoot^-1^) | 2 | - | - | - |
|  | 3 | 0.036 ± 0.007^a^ | 0.065 ± 0.008^b^ | 0.068 ± 0.008^b^ |
|  | 4 | 0.091 ± 0.016^a^ | 0.19 ± 0.04^ab^ | 0.27 ± 0.13^b^ |
|  | 5 | 0.24 ± 0.07^a^ | 0.35 ± 0.11^ab^ | 0.49 ± 12^b^ |
|  | 6 | 0.52 ± 0.16^a^ | 0.68 ± 0.13^a^ | 1.3 ± 0.3^b^ |
|  | 8 | 1.4 ± 0.4^a^ | 2.1 ± 0.3^a^ | 3.4 ± 0.7^b^ |

For mycorrhization conditions, see Table 1. The data are means of four plants ± SD. Values with different letters in each row are significantly different across treatments according to one-way ANOVA followed by Student-Newman-Keuls test (*P*<0.05). wpi, weeks *post* inoculation.
