# Supplementary material for: Mycorrhiza Symbiosis Increases the Surface for Sunlight Capture in Medicago truncatula for Better Photosynthetic Production
Source: PLoS One. 2015 Jan 23;10(1):e0115314. doi: 10.1371/journal.pone.0115314 (PMC4304716; doi:10.1371/journal.pone.0115314)
Supplement: S2 Table — For mycorrhization conditions, see Table 1. The data are means of four plants ± SD. Values with different letters in each row are significantly different across treatments according to one-way ANOVA followed by Student-Newman-Keuls test (P<0.05). wpi, weeks post inoculation. (DOCX) [file pone.0115314.s006.docx]

**Table S2. Shoot developmental parameters of control, mycorrhized (AM) and phosphate-fertilized (P_i_) plants*.***

| **Parameter** | **wpi** | **Control** | **AM** | **P_i_** |
| --- | --- | --- | --- | --- |
| Number of metamers | 2 | 4.8 ± 0.5^a^ | 5.0 ± 0.8^a^ | 5.0 ± 0.8^a^ |
|  | 3 | 14 ± 2^a^ | 21 ± 3^b^ | 21 ± 2^b^ |
|  | 4 | 33 ± 8^a^ | 50 ± 6^ab^ | 68 ± 26^b^ |
|  | 5 | 64 ± 14^a^ | 81 ± 16^a^ | 120 ± 30^b^ |
|  | 6 | 120 ± 20^a^ | 130 ± 10^a^ | 230 ± 60^b^ |
|  | 8 | 270 ± 50^a^ | 270 ± 30^a^ | 650 ± 90^b^ |
| Number of primary branches | 2 | 0 ± 0^a^ | 0.75 ± 0.50^a^ | 0.25 ± 0.50^a^ |
|  | 3 | 2.8 ± 0.5^a^ | 4.0 ± 0^b^ | 3.8 ± 0.5^b^ |
|  | 4 | 4.6 ± 0.5^a^ | 5.0 ± 0.0^a^ | 6.8 ± 1.9^a^ |
|  | 5 | 5.3 ± 0.5^a^ | 6.0 ± 0.9^a^ | 8.8 ± 1.0^b^ |
|  | 6 | 7.8 ± 0.5^a^ | 8.8 ± 1.0^a^ | 9.5 ± 1.3^a^ |
|  | 8 | 9.5 ± 0.6^a^ | 9.5 ± 1.3^a^ | 13 ± 1^b^ |
| Length of primary branches (cm) | 2 | 0 ± 0^a^ | 0.38 ± 0.25^a^ | 0.13 ± 0.25^a^ |
|  | 3 | 0.73 ± 0.30^a^ | 3.3 ± 0.5^c^ | 2.5 ± 0.4^b^ |
|  | 4 | 8.7 ± 4.2^a^ | 22 ± 4^b^ | 25 ± 12^b^ |
|  | 5 | 27 ± 8^a^ | 46 ± 14^b^ | 55 ± 11^b^ |
|  | 6 | 67 ± 26^a^ | 89 ± 7^ab^ | 120 ± 20^b^ |
|  | 8 | 130 ± 10^a^ | 200 ± 30^b^ | 220 ± 30^b^ |

For mycorrhization conditions, see Table 1. The data are means of four plants ± SD. Values with different letters in each row are significantly different across treatments according to one-way ANOVA followed by Student-Newman-Keuls test (*P*<0.05). wpi, weeks *post* inoculation.
