# Supplementary material for: Mycorrhiza Symbiosis Increases the Surface for Sunlight Capture in Medicago truncatula for Better Photosynthetic Production
Source: PLoS One. 2015 Jan 23;10(1):e0115314. doi: 10.1371/journal.pone.0115314 (PMC4304716; doi:10.1371/journal.pone.0115314)
Supplement: S3 Table — The pigments were extracted using methanol and quantified spectrophotometrically. The data are expressed per leaf area and are means of four plants ± SD. There is no significant difference across treatments (at P<0.05). For mycorrhization conditions, see Table 1. wpi, weeks post inoculation. (DOCX) [file pone.0115314.s007.docx]

**Table S3. Leaf chlorophyll and carotenoid content of control, mycorrhized (AM) or P_i_*-*fertilized plants.**

| **wpi** | **Chlorophyll *a+b* (µg cm^-2^)** | | |  | **Chl *a/b*** | | |  | **Carotenoids (µg cm^-2^)** | | | |  |
| --- | --- | --- | --- | --- | --- | --- | --- | --- | --- | --- | --- | --- | --- |
|  | **Control** | **AM** | **P_i_** |  | **Control** | **AM** | **P_i_** |  | | **Control** | **AM** | **P_i_** | |
| 3 | 49.73 ± 5.27 | 47.34 ± 4.23 | 38.63 ± 1.36 |  | 1.94 ± 0.07 | 1.78 ± 0.12 | 1.94 ± 0.06 |  | | 2.05 ± 0.05 | 2.56 ± 0.45 | 2.49 ± 0.03 | |
| 4 | 52.71 ± 2.78 | 52.27 ± 2.00 | 53.12 ± 5.32 |  | 2.50 ± 0.03 | 2.53 ± 0.04 | 2.40 ± 0.08 |  | | 4.47 ± 0.28 | 4.39 ± 0.04 | 4.71 ± 0.37 | |
| 5 | 50.33 ± 1.90 | 53.68 ± 1.92 | 51.72 ± 1.62 |  | 2.39 ± 0.03 | 2.48 ± 0.07 | 2.39 ± 0.05 |  | | 4.55 ± 0.20 | 4.55 ± 0.09 | 4.38 ± 0.29 | |
| 6 | 51.73 ± 1.39 | 55.69 ± 2.80 | 52.40 ± 1.60 |  | 2.49 ± 0.04 | 2.36 ± 0.04 | 2.39 ± 0.06 |  | | 4.73 ± 0.23 | 5.73 ± 0.48 | 4.40 ± 0.31 | |
| 8 | 58.15 ± 2.23 | 60.89 ± 5.95 | 64.41 ± 6.29 |  | 2.30 ± 0.04 | 2.28 ± 0.04 | 2.44 ± 0.04 |  | | 4.60 ± 0.31 | 4.19 ± 0.35 | 3.12 ± 1.00 | |

The pigments were extracted using methanol and quantified spectrophotometrically. The data are expressed per leaf area and are means of four plants ± SD.

There is no significant difference across treatments (at *P*<0.05). For mycorrhization conditions, see Table 1. wpi, weeks *post* inoculation.
